# Supplementary material for: Angle-Dependent Raman Scattering Studies on Anisotropic Properties of Crystalline Hexagonal 4H-SiC
Source: Materials (Basel). 2022 Dec 8;15(24):8751. doi: 10.3390/ma15248751 (PMC9781583; doi:10.3390/ma15248751)
Supplement: Supplementary file 1 [file materials-15-08751-s001.zip › materials-2041072-supplementary.pdf]

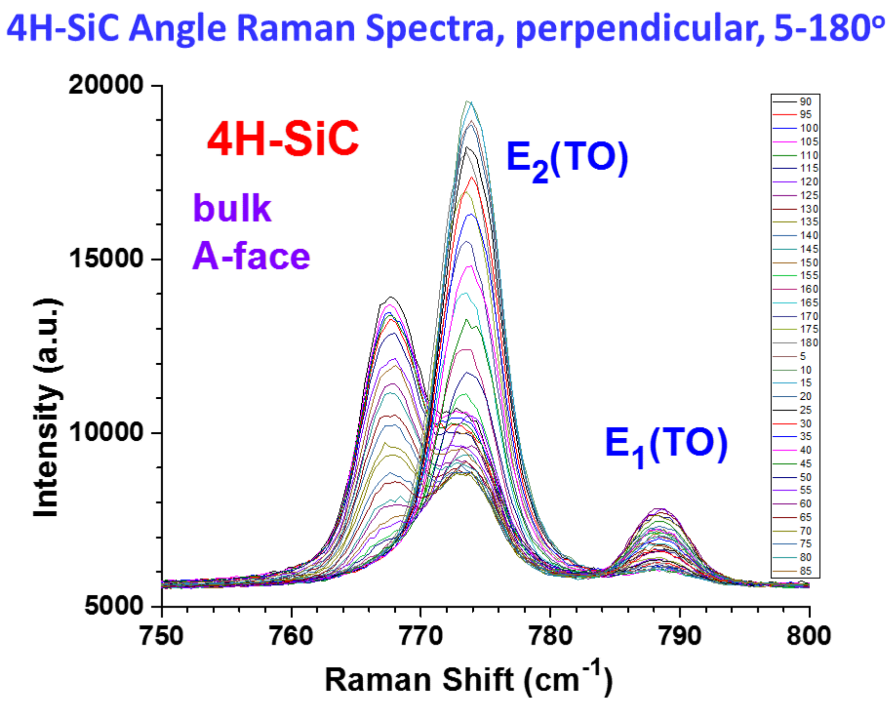

Figure S1. Raman spectrum of the a-face 4H-SiC, including an inset and showing E_1(TA) at 266 cm^{-1}, E_1(TA) at 610 cm^{-1}, E_2(TO) at 775 cm^{-1}, E_1(TO) at 788 cm^{-1}, A_1(LO) at 976 cm^{-1} and LO-plasma coupling (LOPC) broad band in the right side of A_1(LO) [17,18].
